# Evaluation of setup and intrafraction motion for surface guided whole‐breast cancer radiotherapy

**DOI:** 10.1002/acm2.12599

**Published:** 2019-06-11

**Authors:** Sandra Helene Hattel, Peter Andreas Andersen, Isak Hannes Wahlstedt, Sidsel Damkjær, Arpit Saini, Jakob Borup Thomsen

**Affiliations:** ^1^ Department of Electrical Engineering Technical University of Denmark Lyngby Denmark; ^2^ Department of Oncology Section of Radiotherapy Rigshospitalet, Copenhagen Denmark; ^3^ Department of Clinical Oncology and Radiotherapy Zealand University Hospital Næstved Denmark

**Keywords:** breast cancer, intrafractional movement, optical surface imaging, setup accuracy, surface guided radiotherapy

## Abstract

Surface Guided Radiotherapy (SGRT) is a relatively new technique for positioning patients and for monitoring patient movement during treatment. SGRT is completely non‐invasive since it uses visible light for determining the position of the patient surface. A reduction in daily imaging for patient setup is possible if the accuracy of SGRT is comparable to imaging. It allows for monitoring of intrafraction motion and the radiation beam can be held beyond a certain threshold resulting in a more accurate irradiation. The purpose of this study was to investigate setup uncertainty and the intrafraction motion in non‐gated whole breast cancer radiotherapy treatment using an integrated implementation of AlignRT (OSMS) system as SGRT. In initial setup, SGRT was compared to three‐point setup using tattoos on the patient and orthogonal kV imaging. For the investigation of intrafraction motion, OSMS monitored the patient with six degrees of freedom during treatment. Using three‐point setup resulted in a setup root‐mean‐square error from the isocenter of 5.4 mm. This was improved to 4.2 mm using OSMS. For the translational directions, OSMS showed improvements in the lateral direction (*P* = 0.0009, Wilcoxon rank‐sum), but for the longitudinal direction and rotation it was not possible to show improvements (*P* = 0.96 and *P* = 0.46, respectively). The vertical direction proved more accurate for three‐point setup than OSMS (*P* = 0.000004). Intrafraction motion was very limited with a translational median of 1.1 mm from the isocenter. While OSMS showed marked improvements over laser and tattoo setup, the system did not prove accurate enough to replace the daily orthogonal kV images aligned to bony anatomy.

## 1 INTRODUCTION

The positioning of breast cancer patients prior radiotherapy has traditionally been performed by employing three tattoos on the thorax of the patient and a laser system in the treatment room aligned to the accelerator isocenter. The introduction of image guided radiotherapy (IGRT) has enabled the use of smaller margins to reduce treatment‐related toxicity while still ensuring adequate dose coverage of the target volume. However, IGRT usually comes with an extra dose to the patient due to the acquisition of planar kilo‐voltage (kV) images or three‐dimensional cone‐beam computed tomography (CBCT). Recently, surface guided radiotherapy (SGRT) has become commercially available.^1^ It employs visible light for determining the position of the patient surface and is therefore completely non‐invasive. Potentially, a reduction in imaging dose for patient setup is possible if the accuracy of the SGRT systems are comparable to x‐ray imaging. Furthermore, the intrafraction movement can be monitored and the radiation beam interrupted beyond a certain threshold resulting in a more accurate irradiation. It is obvious that positioning based on soft tissue on the surface of the patient differs fundamentally from using internal structures in x‐ray images. However, for tumors located close to the surface, SGRT is expected to be comparable to IGRT and could in some cases be an even better choice. Breast cancer radiotherapy is an appropriate application for SGRT because the target is the breast tissue for which bony anatomy on x‐ray images is a poor surrogate for positioning of the breast. Second SGRT also enables correction of any deformations of the breast tissue resulting in a more accurate dose deposition.^2^ Third, SGRT allows avoiding dose from imaging, particular important for this patient group with a relatively young age profile and good prognosis. Lastly, the possibility to avoid tattoos on the patient for setup also count as an advantage.

Some investigations on the accuracy of the surface systems from different vendors have already been performed for breast patients with different results.^3^, ^4^, ^5^, ^6^ Typically, an improvement in positioning is reported compared to the three‐point patient setup but with a significant difference compared to x‐ray images. Intrafraction motion during radiotherapy has also been investigated and reported to be within the usual treatment margins of the order of 5 mm.^7^, ^8^

In this study we employ SGRT for positioning patients treated with whole breast irradiation in free breathing. We compare setup strategies using either three‐point localization with tattoos on the thorax of the patient or SGRT followed by orthogonal kV images. We aimed for positioning the breast deformed in the same manner as at the time of planning, that is, with the entire breast as match surface for the system. The importance of reproducing the breast deformation is relevant for complex IMRT plans.^2^ Furthermore, for setup of partial breast treatments it may be crucial to have a high reproducibility of the breast position and deformation. In addition to setup by SGRT, all treatments were monitored using the surface system to evaluate the importance of intrafraction motion.

## 2 METHODS

The Varian optical surface monitoring system (OSMS) used for this study (Varian Medical Systems, Palo Alto, CA, USA) is an integrated implementation of the AlignRT surface imaging system (VisionRT, London, UK) on the Varian Truebeam accelerator. It uses a visible light speckle pattern projected on the patient for determining the position of a region‐of‐interest (ROI) on the patient surface.^1^, ^9^, ^10^ For the ROI, the actual position is compared to the ideal position from the planning CT and a rigid deformation is calculated to assist the patient setup. Furthermore, OSMS has the option to monitor the patient surface during treatment and interrupt the beam when a user‐defined threshold is exceeded.

**Figure 1:**
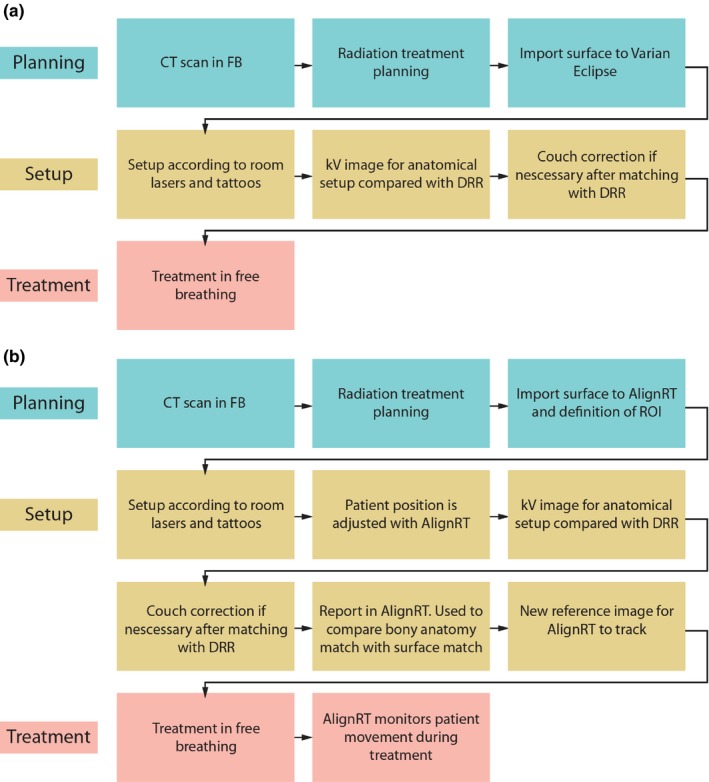
(a) The workflow for the patient group where only room lasers and tattoos were used for positioning. (b) The workflow for the patient group where room lasers and tattoos were used for setup and OSMS was used for adjusting the patient position

In this study, ten consecutive patients referred to postoperative breast irradiation following lumpectomy were investigated. Out of ten, two patients received partial breast irradiation while the rest had whole breast radiotherapy. As explained below, all patients are set up as if the entire breast were to be treated and therefore effectively this is a study of whole breast patient positioning. Age ranged from 54 to 89 yr with a mean of 71.4 yr and with breast size ranging from 190 to 734 cm^3^ with a mean of 426 cm^3^. All patients underwent a free‐breathing planning CT on a Philips scanner. The patients were stabilized in a breast board (Conchest, Candor, Denmark) with both arms above the head for planning and treatments. The planning CT was used for setup of treatment fields in (Varian Eclipse, Varian Medical Systems, Palo Alto, CA, USA) and for dose calculation. The surface of the patient was generated from the treatment planning system threshold (−350 HU), exported to OSMS and a ROI including the entire breast with a margin of approximately 2 cm was used. Quality assurance of the OSMS was performed daily with a phantom provided by the vendor to test x‐ray vs OSMS isocenter coincidence. Most of the days patients were aligned using both three‐point setup and surface guidance, the other days the patients were only aligned using three‐point setup due to logistic reasons. In all cases, the patient position was verified using orthogonal kV images and these were used as our current standard to evaluate the deviations in the two other setup strategies. In the kV images, columna was used as primary match structure with sternum and thorax wall as secondary.^11^ In Fig. 1(a), the schematic workflow illustrates the laser setup, whereas Fig. 1(b) illustrates the corresponding workflow when OSMS is used. For the evaluation of the intrafraction motion, a reference surface was acquired just before turning the treatment beam on. OSMS monitored the patient surface during the entire treatment fraction with a sampling frequency of 2–3 Hz depending on the size of the ROI. From the data file the real‐time data was postprocessed such that deviations from the reference surface were analyzed only when the beam was on.

## 3 RESULTS AND DISCUSSION

**Figure 2:**
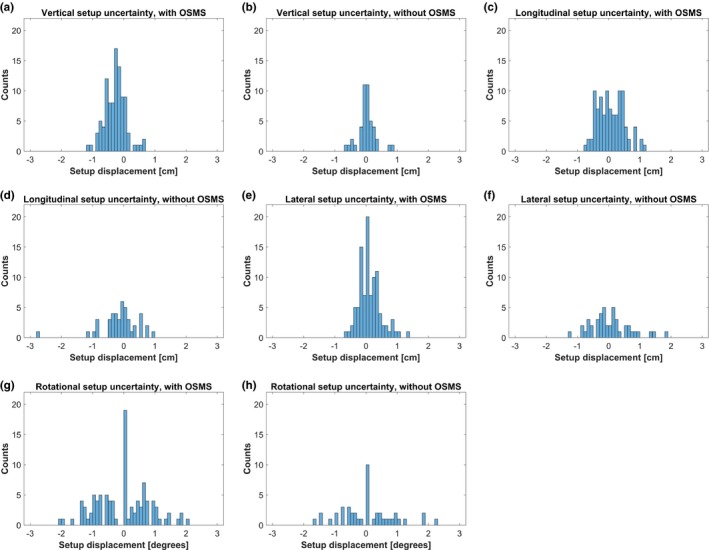
The residual translational and rotational setup error when using OSMS (a), (c), (e), (g) and without OSMS (b), (d), (f), (h)

In total, the study includes data from 143 fractions out of which OSMS was used for 99 fractions. In Fig. 2, histograms of the residual setup error are shown for the two setup strategies. The residual setup error for OSMS in the three spatial directions and rotation corresponding to rotation of the couch is shown in Fig. 2 (a, c, e, g). The corresponding histograms when only room lasers are used, are shown in Fig. 2 (b, d, f, h). A Kolmogorov‐Smirnov test has been used to test data for normal distribution.

Comparison between the two setup strategies was performed with the Wilcoxon rank sum test. We tested the data for equal distributions of the residual root‐mean‐square (RMS) displacement with a significance level of 5%. For the longitudinal displacement and rotational deviation, there was no significant advantage in using OSMS compared to three‐point setup (*P* = 0.96 and *P* = 0.46, respectively). The lateral direction was significantly better determined with OSMS (*P* = 0.0009). Interestingly, the vertical direction was significantly better determined using three‐point setup than OSMS (*P* = 0.000004). Both setup methods include the use of three‐point setup. Therefore, any systematic error from the tattoos could potentially be present in both samples bringing the two samples closer together. From Fig. 2(a) it can be observed that the distribution is not centered around zero displacement, that is, a systematic setup error in the vertical direction is introduced when using OSMS. This could be caused by the patients being more relaxed at the treatment sessions compared to the planning CT. When using OSMS, a compensation for a lower thorax surface is accomplished by a couch shift upwards. Afterwards, an OBI match on columna will reveal a too high position in accordance with the results shown in Fig. 2(a). On the other hand, tattoos on the side of the patient are less sensitive to this effect as observed in Fig. 2(b). This is not a weakness of OSMS, but rather a consequence of the difference in match strategy compared to x‐ray imaging setup. Comparing the OSMS setup with the kV imaging, a 3D residual setup uncertainty of 4.2 mm RMS remains.

**Figure 3:**
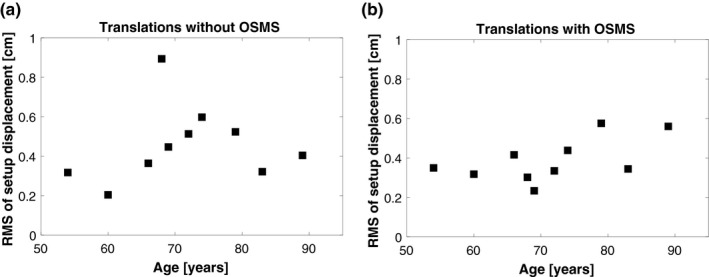
RMS of setup displacement for each patient sorted in ascending patient age. (a) is without OSMS and (b) is with OSMS

**Figure 4:**
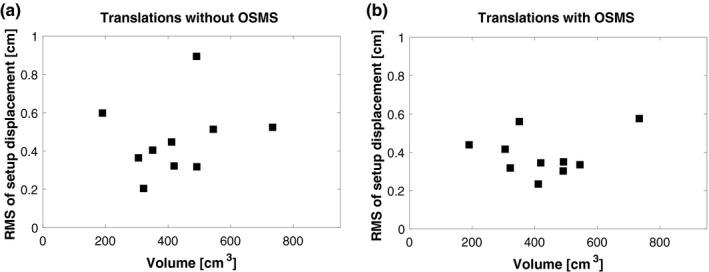
RMS of setup displacement for each patient sorted in ascending breast volume. (a) is without OSMS and (b) is with OSMS

Because we use kV imaging and bony anatomy as comparison to breast position, it could be hypothesized that the accuracy and the mobility of the breast are correlated. We analyzed if setup accuracy correlated with breast size or age for both methods of setup, Figs. 3 and 4. Figure 3(a) indicates that there could be a dependency between setup accuracy and the patient age for the tree‐point localisation setup. However, the small sample size of this study prevents any further investigations. Regarding the OSMS setup method, Fig. 3(b), there may be some dependency, but this is not evident. In Fig. 4 the dependency on breast volume is showed, and a clear trend is not evident for neither.

Compared to previous published results we report setup accuracies in the same range even though we use a region‐of‐interest including the entire breast. Stanley et al. compared SGRT with CBCT and report 3D residual setup uncertainties of 6 mm.^4^ Walter et al. found residual setup uncertainties of about 5 mm.^5^ Both studies showed an improvement compared to tattoo setup. Our results are therefore in good correspondence with these and confirm that alignment of the soft tissue is feasible.

**Figure 5:**
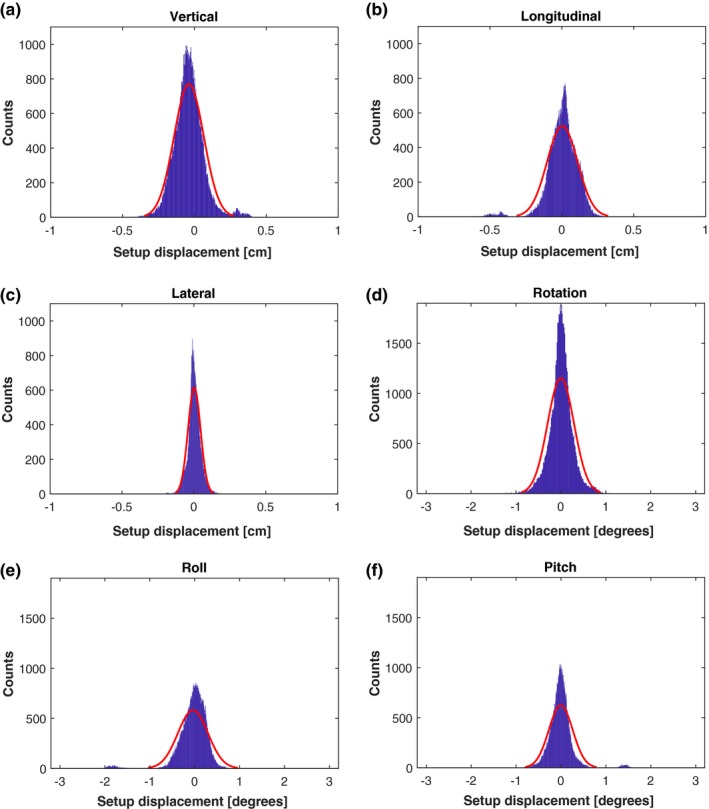
The intrafractional motion of all patients during 99 fractions in all six degrees of freedom. The red curve represents a normal distribution fit

In Fig. 5, histograms of the intrafraction motion along with rotational deviations are shown. It is seen that the intrafraction motion is approximately normal distributed around zero and with only a minor fraction of observations above 3 mm in all three directions. Total amount the observations exceeded 3 mm for vertical, longitudinal and lateral directions were 1.8%, 1.8%, and 0%, respectively. Especially the lateral position seems to be very stable during treatment. The median of the intrafraction translational motion is 1.1 mm whereas the median of rotational deviations is smaller than 0.4 degrees. This is slightly smaller than other studies report using cine portal imaging.^12^ This difference may be explained by the fact that we are comparing the current surface with a reference acquired just before treating, while cine imaging usually compares with a digital reconstructed radiograph obtained from the planning CT. Another limitation of cine imaging is the 2‐dimensional nature while a surface system locates 3 dimensions at the same time. In another study, cine portal imaging was used for relative measurements to get a more realistic intrafraction motion and they report intrafraction motion to be less than 1 mm and comparable to our results.^13^ A study by Reitz et al. used the C‐rad SGRT system (Uppsala, Sweden) for monitoring intrafraction motion in a large cohort and reported a median deviation vector of 1.63 mm.^7^ This result is slightly larger than what we found but could be explained by the small population size of our study, different region‐of‐interest and a different system. In comparison with OSMS, the C‐rad system uses a non‐rigid algorithm which can account for deformations of the surface. The breast is a highly deformable anatomical region and therefore the rigid registration that the OSMS calculations uses may not detect local deformations in breast size and/or position during patient setup. On the other hand, in monitoring the patient the rigid algorithm will not play an important role since a new surface reference is captured after patient setup for OSMS to track during treatment and our results show that the surface stays very stable. The advantage of a non‐rigid algorithm is therefore more or less limited to the positioning prior treatment. The intrafraction motion on the order of 1 mm is well below the difference between using SGRT and x‐ray for setup and therefore a confirmation that the difference is not due to for example, acquiring images in different parts of the breathing pattern.

## 4 CONCLUSIONS

The purpose of this study was to evaluate OSMS as a surface scanner in positioning and monitoring whole breast cancer patients planned for radiation therapy.

A translational residual setup uncertainty was found for tattoos alone to be 5.4 mm. This was improved to 4.2 mm using OSMS. However, while this might seem advantageous the residual setup uncertainty of 4.2 mm remains and OSMS is not accurate enough to replace the daily kV images aligned to bony anatomy. This is in line with previous studies which also conclude that OSMS should be followed by image‐guided setup. Intrafraction motion was very limited with a translational median of 1.1 mm from the isocenter. Based on earlier studies and the results described in our study, it can be concluded that OSMS can be a useful auxiliary tool for monitoring gross motion during radiation therapy, as well as being able to assist in the positioning of the patients.

## CONFLICT OF INTEREST

No conflict of interest.
